# Mutations with pathogenic potential in proteins located in or at the composite junctions of the intercalated disk connecting mammalian cardiomyocytes: a reference thesaurus for arrhythmogenic cardiomyopathies and for Naxos and Carvajal diseases

**DOI:** 10.1007/s00441-012-1365-0

**Published:** 2012-03-27

**Authors:** Steffen Rickelt, Sebastian Pieperhoff

**Affiliations:** 1 Helmholtz Group for Cell Biology, German Cancer Research Center (DKFZ), Im Neuenheimer Feld 581, Building TP4, 69120 Heidelberg, Germany; 2 Progen Biotechnik, Heidelberg, Germany; 3 BHF Centre for Cardiovascular Science, The Queen’s Medical Research Institute, University of Edinburgh, 47 Little France Crescent, EH164TJ Edinburgh, Scotland UK

**Keywords:** Arrhythmogenic ventricular cardiomyopathy, Carvajal disease, Composite junction, Desmosomes, Intercalated disk, Naxos disease

## Abstract

In the past decade, an avalanche of findings and reports has correlated arrhythmogenic ventricular cardiomyopathies (ARVC) and Naxos and Carvajal diseases with certain mutations in protein constituents of the special junctions connecting the polar regions (intercalated disks) of mature mammalian cardiomyocytes. These molecules, apparently together with some specific cytoskeletal proteins, are components of (or interact with) composite junctions. Composite junctions contain the amalgamated fusion products of the molecules that, in other cell types and tissues, occur in distinct separate junctions, i.e. desmosomes and adherens junctions. As the pertinent literature is still in an expanding phase and is obviously becoming important for various groups of researchers in basic cell and molecular biology, developmental biology, histology, physiology, cardiology, pathology and genetics, the relevant references so far recognized have been collected and are presented here in the following order: desmocollin-2 (Dsc2, DSC2), desmoglein-2 (Dsg2, DSG2), desmoplakin (DP, DSP), plakoglobin (PG, JUP), plakophilin-2 (Pkp2, PKP2) and some non-desmosomal proteins such as transmembrane protein 43 (TMEM43), ryanodine receptor 2 (RYR2), desmin, lamins A and C, striatin, titin and transforming growth factor-β3 (TGFβ3), followed by a collection of animal models and of reviews, commentaries, collections and comparative studies.

**Table 1:** Desmosomal proteins

Protein	References presented in alphabetical and year order	
Desmocollin-2	Heuser et al. 2006	Christensen et al. 2010a
	Syrris et al. 2006b	Cox et al. 2010
		de Bortoli et al. 2010
	Beffagna et al. 2007	Xu et al. 2010
	
	Bhuiyan et al. 2009	Gehmlich et al. 2011a
	Simpson et al. 2009	
Desmoglein-2	Awad et al. 2006a	Christensen et al. 2010a
	Pilichou et al. 2006	Cox et al. 2010
		Gehmlich et al. 2010
	Syrris et al. 2007	Xu et al. 2010
		Fressart et al. 2010
	Posch et al. 2008	
	Yu et al. 2008	Gehmlich et al. 2011b
		Kapplinger et al. 2011
	Bhuiyan et al. 2009	Lahtinen et al. 2011
	den Haan et al. 2009	Nakajima et al. 2011
		Sato et al. 2011
	
Desmoplakin	Norgett et al. 2000	den Haan et al. 2009
	
	Rampazzo et al. 2002	Bolling et al. 2010
		Christensen et al. 2010a
	Alcalai et al. 2003	Cox et al. 2010
		Mahoney et al. 2010
	Bauce et al. 2005	Sen-Chowdhry et al. 2010b
	Norman et al. 2005	Xu et al. 2010
	Sen-Chowdhry et al. 2005	Fressart et al. 2010
	
	Norgett et al. 2006	Gehmlich et al. 2011a
	Uzumcu et al. 2006	Lahtinen et al. 2011
	Yang et al. 2006	
		Gomes et al. 2012
	Sen-Chowdhry et al. 2007b	
	
Plakoglobin	McKoy et al. 2000	Asimaki et al. 2009
		den Haan et al. 2009
	Protonotarios et al. 2001	
		Christensen et al. 2010a
	Protonotarios et al. 2002	Xu et al. 2010
	
	Kaplan et al. 2004	Asimaki et al. 2011
		Gehmlich et al. 2011a
	Antoniades et al. 2006	D. Li et al. 2011
	Garcia-Gras et al. 2006	
		Munkholm et al. 2012
	Asimaki et al. 2007	
	
	Huang et al. 2008	
	
Plakophilin-2	Gerull et al. 2004	Otterspoor et al. 2007
	
	Antoniades et al. 2006	Aneq et al. 2011
	Awad et al. 2006b	Adachi and Isobe 2011
	Calkins 2006	Gandjbakhch et al. 2011
	Dalal et al. 2006	Gehmlich et al. 2011a
	Kannankeril et al. 2006	Kapplinger et al. 2011
	Nagaoka et al. 2006	Larsen et al. 2011
	Syrris et al. 2006a	Nakajima et al. 2011
	van Tintelen et al. 2006	Ostrowska Dahlgren et al. 2011
		Pamuru et al. 2011
	Joshi-Mukherjee et al. 2008	Paul et al. 2011
	Lahtinen et al. 2008	van der Zwaag et al. 2011a
	Ram and Van Wagoner 2008	Zhang et al. 2012
	Tandri et al. 2008	
	
	Bhuiyan et al. 2009	
	den Haan et al. 2009	
	Fidler et al. 2009	
	Hall et al. 2009	
	Qiu et al. 2009	
	Watkins et al. 2009	
	Wu et al. 2009	
	
	Christensen et al. 2010a	
	Christensen et al. 2010b	
	Cox et al. 2010	
	Xu et al. 2010

**Table 2:** Non-desmosomal proteins

Protein	References presented in alphabetical and year order
Transmembrane protein 43 (TMEM43)	Merner et al. 2008
	Christensen et al. 2010b
	Christensen et al. 2011
	Klauke et al. 2011
	Larsen et al. 2011
	Pamuru et al. 2011
	
Ryanodine receptor 2 (RYR2)	Rampazzo et al. 1995
	Tiso et al. 2001
	Milting et al. 2006
	Koop et al. 2008
	
Desmin	Klauke et al. 2010
	Otten et al. 2010
	van Spaendonck-Zwarts et al. 2010
	McLendon and Robbins 2011
	
Lamin A/C	Quarta et al. 2011
	
Striatin	Meurs et al. 2010
	
Titin	Taylor et al. 2011
	
TGFβ3	Beffagna et al. 2005

**Table 3:** Animal models

References presented in alphabetical and year order	
Bierkamp et al. 1996	Martin et al. 2009
Ruiz et al. 1996	McCauley and Wehrens 2009
	Pilichou et al. 2009
Gallicano et al. 2001	
	Meurs et al. 2010
Grossmann et al. 2004	
	Fabritz et al. 2011
Kirchhof et al. 2006	Krusche et al. 2011
Meurs et al. 2006	J. Li et al. 2011
Pilichou et al. 2006	Lombardi et al. 2011
	Pilichou et al. 2011
Meurs et al. 2007	
Oxford et al. 2007	Gomes et al. 2012
	Swope et al. 2012
	Kant et al. 2012
Oyama et al. 2008

**Table 4:** Reviews, commentaries, collections and comparative studies published since 2000 reporting that certain mutations in human genes encoding desmosomal proteins and glycoproteins contribute to arrhythmogenic ventricular cardiomyopathies (ARVC) or Naxos and Carvajal diseases

References presented in alphabetical and year order	
Corrado et al. 2000	Casolo et al. 2004
Marcus 2000	Protonotarios and Tsatsopoulou 2004
	Tomé Esteban et al. 2004
Corrado et al. 2001	
Franz et al. 2001	Basso and Thiene 2005
Gemayel et al. 2001	Dokuparti et al. 2005
McRae et al. 2001	Haverkamp et al. 2005
	Navarcikova et al. 2005
Kimura et al. 2002	Schultheiss et al. 2005
Lee et al. 2002	Sen-Chowdhry et al. 2005
Wichter et al. 2002	Thiene et al. 2005
	Wichter et al. 2005
Ahmad 2003	
Bluemke et al. 2003	Basso et al. 2006
di Cesare 2003	Calabrese et al. 2006
Paul et al. 2003	MacRae et al. 2006
	Rampazzo 2006
Bazzi and Christiano 2007	Sen-Chowdhry and McKenna 2006
Marcus et al. 2007	Tsatsopoulou et al. 2006
Pereira et al. 2007	
Sen-Chowdhry et al. 2007a	Delmar and McKenna 2010
Sen-Chowdhry et al. 2007b	Elger et al. 2010
Thiene et al. 2007	Ellinor et al. 2010
van Tintelen et al. 2007	Fressart et al. 2010
	J. Li and Radice 2010
Awad et al. 2008	Lombardi and Marian 2010
Inama et al. 2008	Maass 2010
Whyte et al. 2008	Marcus et al. 2010
	Migliore et al. 2010
Asimaki et al. 2009	Murphy et al. 2010
Bolling and Jonkman 2009	Neuber et al. 2010
Corrado et al. 2009	Pieperhoff et al. 2010
El Demellawy et al. 2009	Remme and Bezzina 2010
Gandjbakhch et al. 2009	Roberts et al. 2010
Hamilton 2009	Sen-Chowdhry et al. 2010a
Herren et al. 2009	Xu et al. 2010
Mackey-Bojack et al. 2009	
Marcus and Zareba 2009	Asimaki and Saffitz 2011
McCauley and Wehrens 2009 ^a^	Asimaki et al. 2011
Saffitz 2009	Avramides et al. 2011
Saffitz et al. 2009	Azaouagh et al. 2011
Sheikh et al. 2009	Basso et al. 2011
van der Zwaag et al. 2009 ^b^	Corrado et al. 2011
	Ghosh and Haddad 2011
Andrews et al. 2010	Lahtinen et al. 2011
Barahona-Dussault et al. 2010	Lombardi and Marian 2011
Bauce et al. 2010	Mezzano and Sheikh 2011
	Palmisano et al. 2011
	Smith 2011
	van der Zwaag et al. 2011b
	
	Pieperhoff 2012

^a^Animal model review

^b^This review contains information on a database from clinical research and other types of data on variants in genes causing arrhythmogenic right ventricular dysplasia/cardiomyopathy (ARVD/C)
